# Mr. Ring, on the Diffusion of Vaccination

**Published:** 1804-11-01

**Authors:** John Ring

**Affiliations:** New Street, Hanover Square


### *To the Editors of the Medical and Physical Journal.*

GENTLEMEN,

IN your Journal for May, 1803, you have given some account of the introduction of vaccine inoculation into Greece, and several parts of Asia; an account of its introduction into Turkey had been given long before. Having about ten months ago received from Dr. De Carro a work, entitled the History of Vaccination in Turkey, Greece, and the East Indies, I embrace the earliest opportunity afforded by other avocations, of transmitting a concise analysis of that valuable and interesting publication. It is, indeed, particularly interesting to Britons, since it contains additional evidence, that their countrymen have been the happy instruments of diffusing the blessings of Vaccination over a considerable part of the globe.

Dr. De Carro dedicated his first publication on this subject to Lord Minto, at that time the British Ambassador at the Court of Vienna; the present he dedicates to the Hon. Arthur Paget. These marks of respect he considered as due to the British Nation; the first, because it relates to a discovery, for which the world is indebted to this nation; the second, because it relates to a quarter of the world, in which she has so far extended her empire, and the glory of her arms.

Dr. De Carro observes, that, whether owing to the want of a good understanding between the foreign physicians settled at Constantinople, or to the difficulty of making the Turks sensible of the advantages of vaccination; the first successful experiment, in the family of Lord Elgin, did not produce so much effect as might have been expected. The practice was repeatedly discontinued, and only renewed by the arrival of fresh matter, till it was adopted by Dr. Hesse, a German physician.

This gentleman went to Constantinople, with the intention of settling there as an oculist; but was on the point of quitting Turkey when he received from Vienna some cow-pock matter; which induced him to alter his plan, thinking it probable that he might succeed better from the practice of vaccination, than from the profession of an oculist.

The first child of the Earl of Elgin was vaccinated by Dr. Whyte, the second by Dr. Scot. These gentlemen also also vaccinated some children in the families of Ambassadors, and other foreigners of distinction; but they were not able to overcome the prejudice of the natives. This was in a great measure reserved for Dr. Hesse, who successfully practiced vaccination in Constantinople for the space of a year and a half.

In the month of March, 1802, the Earl and Countess of Elgin, and Dr. Scot, sailed from Constantinople, with an intention of making a voyage to the Islands of the Archipelago, and the greater part of Greece. During their absence, vaccination was practiced by Dr. Hesse, Dr. Pazzoni, physician to the Spanish Ambassador, and Dr. Auban, physician to the French Ambassador: but on the departure of Dr. Hesse, it was suffered to become extinct.

"Our voyagers," says Dr. De Carro, "did not neglect vaccination during their stay in those celebrated countries." The following is an extract of a letter which he received from Dr. Scot on this subject.

"You will be much pleased to hear, that the blessing of vaccination has been communicated to the celebrated city of Athens, where we have stayed a long time; and that it is probable, it will thence propagate itself through the different parts of Greece.

"Immediately on my arrival, I sent for Dr. Cassgitti, the principal physician of Athens; and, having conversed with him concerning the origin and the character of vaccination, I vaccinated two children in his presence, and succeeded in one of them. This child served to vaccinate a great number; and at the time of our departure, more than eighty persons, of all ages, had been vaccinated with the usual success. Dr. Cassgitti appeared to me to be a judicious and active physician; I made him a present of a copy of your work.

"I also spoke to the principal surgeons of Argos, Corinth, and other celebrated cities, on the subject of vaccination; and although they have still some prejudices to conquer, I doubt not but its advantages will be very generally acknowledged in Greece, as well as every where else.

"But while I inform you of all that we have done during our voyage, you will be surprised to learn, that they have ceased to propagate vaccination at Constantinople. Dr. Hesse, who was the principal vaccinator, being about to leave that country, neglected to keep up a fresh supply of matter. WM. SCOTT."

In

In addition to this account of the introduction of vaccine inoculation into Greece, Dr. Scot himself, when in England, favoured me with the following particulars.

On his arrival at Athens, in April 1802, he had some conversation with Dr. Cassgitti, who was educated at Pavia. The little he knew of vaccination was from the Literary Journals. No such disease in cows had been observed in that country.

The Athenians, who, notwithstanding their present state of subjection, still retain something of the quick and penetrating spirit of their ancestors, were so struck with the mild nature of the cow-pock, when compared with the small-pox, that they eagerly brought their children to partake of the benefit. Fresh matter, with instructions, was sent to Thebes, Corinth, Argos, and other places; for the result of which, as well as the progress of vaccination in Laconia, he referred me to a letter he had received from Dr. Cassgitti, which is now in my possession.

In this letter, dated October 1802, Dr. Casgitti informs Dr. Scott, that he had no more subjects to inoculate. He also gives an account of the progress of vaccination in different parts of Greece.

A brother of Dr. Cassgitti, having introduced vaccination into some of the country parts of Laconia, where the small-pox made great ravages, was induced to set a price on it, in order to reimburse himself for the expences he had incurred; but the artful inhabitants, though rich to an excess, commenced the practice themselves; and propagated it from one village to another, in such a manner, that the practitioner who introduced this novelty, soon discovered he was no longer of any consequence in that neighbourhood.

In my Treatise on the Cow-pox I have related some particulars concerning the introduction of the practice into Salonica; and therefore deem it unnecessary to repeat them here. Dr. La Font informed Dr. De Carro, "that he esteemed himself very happy in having it in his power to pay the City of Salonica the tribute of acknowledgment, which the world owed her, for having been the birth-place of the old woman who practised inoculation at Constantinople, when she attracted the attention of the celebrated Lady Mary Wortley Montague,"

The matter which Dr. La Font received from Dr. De Carro upon ivory lancets, readily succeeded; and all the inhabitants of Salonica adopted vaccination with an eagerness worthy of the most enlightened people. Turks, Armenians, Greeks, Franks, all had recourse to this preservative; and the inhabitants of the neighbouring towns, wishing to enjoy the same blessing, Dr. La Font instructed pupils, in order to spread the practice.

Dr. Moreschi of Venice, who first introduced vaccination into that city and a great part of Italy, and published two works on the subject, also had a considerable share in disseminating the practice through Dalmatia and other provinces, situated on the Adriatic; and through the greatest part of Greece.

Dr. Hesse is said to have been the principal inoculator of the cow-pock at Constantinople; but several other physicians contributed to the same laudable design. Among others, Dr. Roini, physician to the Grand Seignior, printed an extract from Dr. De Carro's Treatise on the subject, translated into the Turkish language, and presented it to his Highness, who had suffered much from the small-pox. He expressed his regret that the discovery had not been made in his youth; and desired it might be adopted in his territories. Dr. Roini vaccinated a child of one of the servants in the Seraglio; but the Turks, who are always enemies to innovations, did not embrace vaccination with any degree of zeal: so that the practice again fell into disuse.

It was again revived in the beginning of the year 1803; when Dr. Auban, physician to the French Ambassador, wrote to Dr. De Carro for some matter; and informed him, that more than a hundred persons, of different nations and religions, waited with impatience for the arrival of this preservative. He added, that up to this period he had only vaccinated one child of any Turk; but that among those ready to undergo the operation, were three children of the first physician of the Grand Seignior.

At the same time Dr. De Carro received a letter from the Earl of Elgin, requesting matter for his third child; the success of which may be learned from the following extract of a letter, which I received from Dr. Scot when in England, dated October 30, 1803.

"I often conversed with Dr. Roini, physician to the Grand Seignior, upon the salutary influence of vaccine inoculation; and the number of lives that might be saved, if it were generally adopted in the Turkish empire. I found him zealous to promote its progress; he recommended it strongly to several of the principal men in power.

"As a little tract, published at Palermo, by Dr. Marshall, appeared to me well adapted to popular use, I gave him him a copy of it; which he got translated into the Turkish language. But notwithstanding all the care taken to encourage vaccination at Constantinople; either from neglect, or want of opportunity, it had fallen into disuse; and, upon my return thither, in October 1802, no matter could be procured.

"I was, therefore, under the necessity of sending to Dr. De Carro at Vienna for a supply. After some failures, it was renewed last January, in the son of the Prussian Minister, and in Lady Harriet Bruce, second daughter of the Earl of Elgin. Recent matter was thereby afforded to different practitioners; and, among others, to Dr. Auban."

This is the gentleman who flattered himself, that he had discovered vaccination to be an antidote for the plague. Dr. Valli, who arrived at Constantinople about that time, coincided in his opinion; and was so far convinced of the reality of this hypothesis, that he inoculated himself with pestilential matter; but he paid dearly for his temerity, by catching the plague, which he did not survive without some difficulty.

Dr. Auban informed Dr. De Carro, that the Armenians have much greater confidence in Vaccination than the Turks. But an ignorant practitioner has damped their ardour, by producing the spurious pock in three of their children; one of whom fell a victim to that disease. Hence the opinion of Dr Auban, that cow-pock matter possessed an anti-pestilential virtue, though visionary, was not without its use; for it served to revive the practice; and the Turks, who had rejected vaccination as an antidote for the small-pox, adopted it as an antidote for the plague.

Dr. Valli is recovered of this disease, and Dr. De Carro informed me in his last letter, that he had seen him at Vienna.

Dr. De Carro is of opinion, that the zeal of our countrymen in Asia, and of the natives, for vaccination, should make those practitioners in England blush, who still inoculate for the small-pox. He is confident, that no children have been inoculated for the small-pox in any part of Germany for two years past. He says, the operation is absolutely forgotten; and thinks it as difficult to find patients who would submit to the operation, as medical men who would perform it.

Having sent Dr. De Carro a copy of the Reverend Mr. Warren's excellent popular publication on the subject of inoculation of the cow-pock, he informed me that they have innumerable addresses of this kind in Germany, published by clergymen of all ranks; and, that although they are in a great measure a copy of each other, they have been of great service to those for whose use they were intended. This is an example worthy of imitation.

Dr. De Carro enjoys the enviable satisfaction of having diffused the blessing of vaccination through a great part of the continents of Europe and Asia. This country is peculiarly indebted to him, for the introduction of the practice into her extensive colonies in the East Indies; and the Directors of the East India Company appear to be sensible of the importance of the service.

An account of the commencement and progress of vaccination in India has already appeared in this and other channels. The matter was sent by an overland dispatch, by way of Constantinople, to Bagdad; where it succeeded in the hands of Dr. Short, physician to Mr. Jones, the English Resident. From this place it was transmitted to Bussora, where it succeeded in the hands of Dr. Milne, physician to Mr. Manesty, the English Resident. From this place it was transmitted to Bombay.

Some particulars of the rise and progress of vaccination in India have already appeared in this and other channels. In that part of the world, the small-pox is more fatal, and population more scanty. It is, however, but justice to our countrymen in India to say, that their zeal and exertions have been commensurate with the occasion.

Dr. Milne not only forwarded matter to Bombay, but also to Persia and Arabia. I shall here insert a very interesting extract of one of his letters to Dr. Scot, dated May 1802, which was communicated by that gentleman to me.

"Within the last month I have inoculated upwards of thirty subjects of different ages; and have both transmitted matter by, and vaccinated, several people belonging to three different vessels, which have been dispatched hence for Bombay. I have also sent materials into Persia, by the way of Bushire; and furnished the means of inoculation to the Resident at Muscat.

"The postscript of your letter, stating that the common diseases of children have been benefited by vaccine inoculation, has added much confidence to the opinion I had formed of its salutary action, from reading Dr. Jenner's book. I have therefore never hesitated to inoculate every person that has been brought to me; and have the pleasure to state, that a child who had been a long time subject to an intermittent attack, accompanied with a dropsical swelling ing of the belly, has been completely cured by vaccination. This case, with some others, I have drawn up, and sent to India."

From Dr. Milne's letter, and from Dr. De Carro's publication, it appears, that the British Residents, and medical officers at Bagdad and Bussora, vie with the governors and medical establishment in India, in their endeavours to promote vaccination.

Dr. De Carro's work is published in French; he is about to publish another edition in the German language; in which an account of vaccination in Moldavia will be added. The Hospodar of Moldavia has sent him a present of a very handsome Indian shawl, in return for his Treatise.

An extract, which I some time ago published in this Journal from Dr. De Carro's work now under consideration, and letters which I have since received from him, concur in proving, that the hypothesis of an anti-pestilential virtue in vaccination may be considered as perfectly exploded. Dr. Struve of Gorlitz, fondly imagined that he had found the practice a preservative against the scarlatina; but this opinion, as well as that once entertained by some persons in England and France, of its being a preservative against the hooping-cough, is totally destitute of foundation.

In the Bombay Gazette for July 2, 1802, is a letter from Drs. Moir and H. Scott, giving an account of the introduction of vaccination into India; and accurately describing the vaccine vesicle as it appears there. By this description, it is evidently of the genuine kind. Due acknowledgments are also made of the services rendered by the Earl of Elgin and Dr. De Carro; to whom the public are under great obligations for transmitting the virus to India.

Dr. De Carro alludes to the case of Count Mottet, which is well known in the annals of vaccination; and observes, that the physicians of Bombay entertain a more correct opinion of the nature of this case, than the generality of medical men in Europe; who think that a person who has had the small-pox cannot have the genuine cow-pock. Dr. De Carro admits it is difficult to give the genuine disease to those who have had the small-pox; but maintains, from his own experience, that although you can but rarely excite the true vaccine vesicle in a person who has had the small-pox, it is not impossible. In this respect he coincides in opinion with Dr. Jenner.

 Several

Several instances of genuine cow-pock after the small-pox are related in my Treatise on the Cow-pock. Of the reality of such cases, no stronger proof can be required, than that matter taken from them should be capable of propagating the genuine disease. One well known case of the kind is that of Mr. Rooke of Jamaica; another is that of Mr. Tanner, published in the fourth volume of the Medical and Physical Journal.

Dr. De Carro wrote to Dr. La Font, desiring to know whether the plague ever attacks those who have the small-pox, or those who have had that distemper. The answer he received was, that the small-pox, whether a person at present labours under the disorder, or has had it a longer or shorter period, is no preservative against the plague. Dr. La Font has known two persons have the plague and the small-pox at the same time. Those who attend people in the plague, have known many instances of this kind.

Dr. La Font says, the plague has not been known to mitigate the small-pox. He observes, that infancy has been thought particularly disposed to receive infection; but at Salonica they have found it quite the reverse. He says, it has been held as a never-failing rule from all antiquity, that when the plague reigned, all other disorders ceased; but in the year 1793, when the plague prevailed there, the small-pox also raged with unexampled fury. Such were its ravages, that the Jewish nation at Salonica, consisting of twelve thousand, lost a sixth part of their number by that single disease.

Dr. Valli, after suffering severely from the plague, and proving in his own person the inefficacy of vaccination as a preventive of that dreadful distemper, went to Smyrna, and recommended inoculation of the small-pox as a preventive. This, Dr. De Carro justly observes, is the more remarkable, since he seems to acknowledge, that the small-pox is not a preservative against the plague but during the short time while it lasts, which is but a few weeks, and cannot be renewed.

Dr. De Carro concludes his work with a letter from Dr. Jenner, dated March 30, 1803. In this letter, Dr. Jenner observes, that as far as he had been able to learn, the species of rot in sheep, described by Dr. De Carro, was totally unknown among the flocks in Great Britain.

On the arrival of Dr. De Carro's intelligence, respecting the inoculation of sheep, I mentioned the circumstance to a nobleman who has a considerable estate in the county of Sussex; who said, he knew of no such disease as that I described.

described. I next inquired of Dr. Jenner, whether he had ever seen or heard of it? to which he answered in the negative. I asked him, whether he had ever taken any particular pains to investigate the subject? He assured me that he had made it a particular object of inquiry; and had frequently questioned the oldest shepherds on this point; but could never hear of such a disease.

I then caused an inquiry to be made of another person of distinction, who has paid particular attention to the improvement of the breed of sheep. His answer was, we have no knowledge of such a disease, as that described by Dr. De Carro and other foreign authors.

Still, however, it appeared to me highly improbable, that a disease which commits such dreadful ravages on the Continent of Europe, should be utterly unknown in Great Britain. I therefore consulted Layard, "On the Contagious Distempers of Horned Cattle," and was so fortunate as to find a reference to Fuller's Exanthemata, a work not so well known as it deserves to be.*

Under the head, "Rittelen, or Chicken-pox," we meet with the following remarks: "Sometimes they come alone; sometimes they have been seen sprinkled among the measles.

"It is said that poultry and turkies are subject to a disease coming out with red pimples, though not many; which soon dry up into scabs; but are not apt to leave scars or marks.

"Swine or hog-pox.

"Chesneau mentions a sort of pustules, *not differing much from the true small-pox. Many people mistake them for the small-pox; but they continue not so long, bring no danger, and leave no marks.* These, he thinks, cause many people to believe they have had the small-pox more than once.

* The following observation, p. 21, though not connected with medical science, is curious and interesting. "I can almost suspect, that our celebrated Sir Isaac Newton might fetch the first hint of his notions of attraction and gravitation, from a little ludicrous Spanish book, entitled, The Man in the Moon."

"For, p. 46, it is said, 'I found by this experiment, that which no philosopher ever dreamed of, viz. that those beings which we call heavy, do not sink towards the centre of the earth as their natural place, but are drawn by a secret property of the globe of the earth, or rather, something within the same; in like sort as the loadstone draweth iron, within the compass of the beams of attraction."

 "He

"He gives them no name; but I believe them to be what we call the swine-pox."

It is a mistake to affirm, that the swine-pox leaves no marks; but I am inclined to believe, with Fuller, that it is a different disease from the chicken-pox, and that the same person sometimes has them both. The swine-pock containing a more opaque fluid than the chicken-pock, it is not so apt to be broken; but turns about the fifth day, and is converted into a yellow scab. The scab of the small-pock is brown.

Fuller adds, "The small-pox, and its spurious sorts, are peculiar to man. Mr. Mather, indeed, in his letter from Boston in New England, says, that Dr. Leigh, in his Natural History of Lancashire, reports, that some cats were known to catch the small-pox; and pass regularly through it. He adds, We have had among us the same occurrence.

"But if we had seen and examined the matter, perhaps it would have been found a very different thing from the small-pox. For in like manner there was, about the year 1710 or 1711, upon the South Downs in Sussex, a certain fever raging epidemically among the sheep, which the shepherds called the small-pox; and truly, in most things, it nearly resembled it.

"It began with a burning heat, and unquenchable thirst. It broke out in fiery pustules all over the body. These pustules maturated; and if death happened not first, dried up into scabs about the twelfth day.

"It could not be cured, no, nor in the least mitigated by phlebotomy, drinks, or any medicines or methods they could invent or hear of.

"It was exceedingly contagious and mortal; for where it came, it swept away almost whole flocks. But yet it could in no wise be accounted the same with our human small-pox; because it never infected mankind."

Ere I quit Fuller, I beg leave to observe, that he mentions an instance of the co-existence of the small-pox and the measles, published by Dr. Ridley. In my Treatise on the Cow-pox I have related a considerable number of instances of the co-existence of these and other eruptive diseases, contrary to the opinion then prevailing. These and almost every thing else of any consequence contained in that publication, a good-natured writer in the Medical Repository, published at New York, has ascribed to Dr. Coxe; although it is evident, by his frequent quotations from my book, that as much of it as was then printed, consisting of 750 pages, was then in his possession. Such misappromisappropriations, however, and such unmerited compliments, are not confined to the other side of the Atlantic; and it would be easy to mention some very vain authors on this side of the water, who, if they were stripped of their borrowed feathers, would be as naked as when they came into the world.

While I was endeavouring to ascertain the nature of the murrain in sheep, Dr. Harrison's valuable Observations on the Rot fell into my hands, and confirmed the opinion I had before entertained, in consequence of the best information I could procure, that what Dr. De Carro describes is a different disease. Of this Dr. De Carro himself is since convinced. He was led into the error of supposing it to be the rot, by the vague manner in which the terms denoting the distempers of sheep are sometimes used.

Dr. Harrison, speaking of the rot, says, "It has been called the sheep-pox by Prof. Vibourg, of the Veterinary College at Copenhagen."

Dr. Harrison is inclined to believe, "That Prof. Vibourg and Dr. De Carro confound the rot with the claveau des moutons; which is a febrile and eruptive disorder. This complaint bears a strong resemblance to the small-pox. The claveau is a vague and indefinite term; it comprises the scab, and rot or pourriture, as well as the disease properly denominated claveau. These are very different affections, and ought not, as I conceive, to be included under one general denomination."

About the period when I perused Dr. Harrison's publication, I met with the passages alluded to in Layard and Fuller; which I thought worthy of being communicated in some popular channel; but, in order to determine that point with the greater precision, I first wrote to Dr. Harrison, to ask if he had ever heard of the sheep-pox in this country.

His letter, dated Nov. 20, 1802, is as follows:

"I was favoured with your letter a few days since, and shall have great pleasure in giving you every information in my power, relative to that curious disorder the claveau des moutons. After many inquiries, I have reason to believe that it is unknown in every part of this island. It differs equally from the rot, or pourriture, which is a complaint of the liver, without any eruption; and from the scab, or la gale, a chronic affection of the skin unattended with fever; though it has been improperly confounded with both.

"Indeed, the complaints of sheep are very little understood; stood; and it gives me great pleasure to find, that you think them worthy of your consideration. My attention was accidentally called to the subject more than twelve months since; and hitherto I have prosecuted the inquiry, principally, from an idea that it will enable me to throw new light upon the disorders of mankind.

"The claveau des moutons is a febrile and an eruptive disease. It resembles the small-pox in so many particulars that I am inclined, from analogy, to believe it may be superseded by cow-pock inoculation. If it were known in this country, I make no doubt that you, to whom the friends of humanity are under so many obligations, would soon favour us with much useful information, concerning the influence of vaccine inoculation over this virulent and dangerous malady.

"Sir Joseph Banks has forcibly pointed out the great danger of importing the claveau into this country, with the Spanish sheep, which our breeders introduce for the improvement of their flocks. It is a subject of national importance, and the public are much indebted to Sir Joseph for the cautions he has recommended.

"If the claveau once makes its appearance in Britain, we may have great difficulty in exterminating it from among us. I think, when sheep are first brought into the island, the importers should be obliged, under severe penalties, to keep them apart from all others, till they are satisfied that no danger is to be apprehended. By attending sufficiently to this precaution, we shall be in less danger of suffering from the ravages of a complaint, which proves fatal to so many sheep upon the European continent, and from which we have hitherto remained free, in consequence, I conceive, of our insular situation.

"I thank you for your obliging inquiries about my little Treatise on the Rot in Sheep, and other Animals. It was inserted in No. 56, of Mr. Young's Annals of Agriculture. I am preparing a new edition of it for the press; which I expect to publish some time next spring.

"I am, &c.

"E. Harrison."

On referring to the Annals of Agriculture, I met with much unexpected information concerning the sheep-pox; but finding that I was anticipated in my intention of being the first to announce that the disease has been known in England, I deferred writing any remarks on the subject, till, till by further investigation, I should be able to add something to the stock of information already acquired.

In the work before mentioned, for the year 1803, p. 631, Sir Joseph Banks has published a caution to the importers of foreign sheep; in which he says, "This dreadful malady having made considerable ravages in many countries on the Continent, and certain individuals having of late years been in the practice of importing Spanish sheep; it well deserves their attention that such a distemper exists, and would, if brought into this country, prove a very serious misfortune.

"Fitzherbert mentions it as known in his time, (*Certain Ancient Tracts concerning the Management of Landed Property,* 1767, *p.* 41) and, under the name of *Claveau.* It is largely treated of in Carlier's *Traitè des Bêtes a Lain,* quarto, vol. ii. p. 519. He describes it as being exceedingly infectious, and fatally destructive to flocks; spreading rapidly over a whole province."

In p. 632 of the same volume of the Annals, are the following observations "On the Pock of Sheep, by Mr. Westfield of Weenda.

"Though I believe I am pretty well acquainted with what has been written by English authors on the subject of husbandry, yet I do not recollect, in any of them, to have met with an account of the pock incident to sheep.

"From this circumstance I concluded that the disease was not to be found in England; and the more so, as a paper in one of the English journals, published about the year 1790, gives an account of the sheep-pock only from French writers on this subject.

"As to the nature of the disease, it bears the greatest resemblance to the small-pox of children. According to what we know and conjecture, it never makes its appearance but from infection; for I have always been able to trace its origin and propagation, in all cases which have fallen under my observation.

"The pocks always make their appearance after a fever, accompanied with a swelling of the glands. They appear as red spots; which gradually undergo suppuration; then they dry, and drop of.

"The disease has three periods; which, however, as to their duration, are not quite regular. From the moment of infection to the eruption of red spots, there are generally seven days. After this period, they require nearly the same space of time to arrive at complete suppuration.

"When this has taken place, the drying and falling off succeed, succeed, after a short space of time; but not seldom the disease terminates in malignant ulcers; which will last for several weeks. As it is no easy matter, in this case, to determine what is the pock itself, or only the consequence of the ulcers produced by it, the duration of this period becomes less certain.

"The sheep-pock is a most ravenous disorder; where a flock is attacked by it, more than half of the sheep are destroyed; and the remaining part is generally unhealthy, and not fit for any farther use. According to my observations, the danger never appears in the two first periods; but constantly in the last, viz. in that of drying and falling off.

"The pustules appear particularly on the naked parts of the body of the sheep; viz. on the belly, between the legs, and in the face. The parts covered with wool are, indeed, not free from being attacked; but such a case is less frequent, and less dangerous.

"Of the naked parts, they appear most frequently in the face; upon and round the eyes and the mouth; as also in the cavity of the mouth, and the nostrils. Those round the eyes are apt to become confluent, and cause very malignant sores; so as to drive the eyes out of their sockets. When they occupy the parts round the mouth, they prevent the animal from taking any nourishment, and cause it to die of hunger.

"Within the nostrils, they cause very considerable inflammation and gangrene. Those subsequent accidents appear to prove more fatal than the original disease; for in most cases the animals seldom die before the twenty-first day, on which the distemper terminates; but often from eight to twenty-one days after this period. Upon dissection, no pustules could be discovered in the internal parts.

"The natural infection was not communicated to other sheep at any great distance; and I am inclined to believe, that it only takes place from immediate contact. My sound flocks remained free from the disease, at the distance of three hundred feet from those which were infected.

"The virus retains its infectious quality a considerable time. A sound sheep introduced into a flock in which the disease had ceased for twenty-nine days, was infected.

"Infection may be produced by different substances taken from the diseased sheep. I have inoculated with bloody lymph, with fresh purulent matter, and with scabs; and in all these cases, the pock was produced. The least quantity quantity of the virus imaginable is sufficient to effect inoculation.

"It is now proved by pretty general observation all over Germany, that inoculation is the most effectual means of preserving the flock; of three hundred and fifty sheep, which I caused to be inoculated with the sheep-pox, not one has died, nor become unhealthy.

"Whether the cow-pock will preserve sheep from the sheep-pock is yet undecided. According to my own observations, the former does not apparently affect sheep; for which reason I have discontinued my experiments."

Having mentioned this object of my inquiry to Major Magra, from whom I received the intelligence I some time ago published in this Journal, concerning the pustulous disease of turkies on the coast of Africa, he told me, that Mr. Elman, who has the care of the famous South-Down flock belonging to Lord Viscount Hampden, is celebrated for his knowledge of the diseases of sheep; and kindly offered to write to the Hon. Mr. Trevor on the subject. From Mr. Trevor's answer, dated Glynd Place, Dec. 16, 1803, I derive the following information.

"There has been no appearance of the disease called small-pox amongst any of the flocks of this county, since 1762 or 1763. In that year most of the flocks in the western part of the county were affected with it, and lost great numbers.

"The symptoms were a great drowsiness, and an eruption of pustules, like those of the small-pox, in the face, and under the arms; which, in those that recovered, filled with a considerable quantity of matter, and afterwards turned to a dry scab. It left marks behind, which were visible as long as the animal lived. The old fat wethers suffered most.

"This disease came from the westward, and was communicated to the neighbouring flocks in the same Downs. Its source was not accurately traced.

"Those flocks suffered least, in which the affected sheep were separated as early as possible from the sound.

"The remedies employed are not now known."

One of the principal objects of this Memoir, was to give an analysis of Dr. De Carro's late publication; and, I apprehend, it will not be deemed inconsistent with that purpose, to add a few extracts from a letter I received from him, dated Vienna, September 8, 1804. It contains a testimonial relative to the Portsmouth cases, similar to those from other respectable quarters.

"Accept

"Accept my best thanks for the pamphlets which you were so good as to send me by Mr. Aveling. I was already acquainted with Mr. Goldson's work. I wish I could shew you the copy which was first sent to me. You would see the marks which I made with a pencil at almost all the same passages where I find your's, in the copy which I received from you.

"My opinion of his performance was, that he had been much too negligent in ascertaining the genuineness of his vaccinations; and that he laid too much stress upon the effects of his variolous inoculations. I looked on those he describes, as merely the consequence of a common cuticular inflammation; such as is produced by most kinds of morbid matter; which sometimes, though rarely, excites a fever.

"It appeared to me, that the whole pamphlet shewed much less information upon vaccination in general, than any man who undertakes to write against a practice sanctioned by the whole world should possess; and I could not help entertaining suspicions concerning the author's partiality for the practice, when I found that so late as the year 1802, he continued to inoculate for the small-pox.

"How any man who understands vaccination, can still continue to practice inoculation for the small-pox, is to me absolutely inconceivable. We see no such thing at Vienna. No practitioner seems to recollect that the inoculation of the small-pox ever existed. If any one wished to revive the practice, it may be doubted whether he could find parents who would permit it; or matter to accomplish his design. I have no doubt, however, but the title of Mr. Goldson's pamphlet must have done some mischief; but I trust, your able answer will diminish its effect.

"You have afforded me the greatest pleasure possible, by giving me an opportunity of reading Mr. Anstey's elegant Latin Ode. It is a rare thing, in our days, to meet with such classical Latin."

After speaking in flattering terms of the translation of that Ode, Dr. De Carro concludes in the following manner; alluding to an expression in my answer to Mr. Goldson, he says, "You will, perhaps, be offended with me; *equo credo; lympham equinam quotidie insero, illamque in ditiones Austriacas, innumerasque alias regiones Europæ et Asiæ, sine metu spargo.*

"Dr. Sacco sent me, last year, two sorts of equine matter; one taken immediately from a horse labouring under *giardoni;* the other from the same source, but already reproduced duced in several human subjects. *Vale, et ama Docteorem De Carro, vaccinatorem et equinatorem; sed nunquam, sicut medici et chirurgi Portûs Magni, variolarum insitorem.*"

I am, &c.

*New Street, Hanover Square.* JOHN RING.

---

