# Errata

**Published:** 1804-12-01

**Authors:** 


### ERRATA.

P. 448, l. 12, for Pazzoni, read Pezzoni.

459, l. 21, for Wecada, read Weende.

463, l. 1, for Docteorem, read Doctorem.

END OF VOL. XII.

